# Final analysis of a phase I/IIa trial of the folate‐binding protein‐derived E39 peptide vaccine to prevent recurrence in ovarian and endometrial cancer patients

**DOI:** 10.1002/cam4.2378

**Published:** 2019-07-05

**Authors:** Tommy A. Brown, Kevin Byrd, Timothy J. Vreeland, Guy T. Clifton, Doreen O. Jackson, Diane F. Hale, Garth S. Herbert, John W. Myers, Julia M. Greene, John S. Berry, Jonathan Martin, John C. Elkas, Thomas P. Conrads, Kathleen M. Darcy, Chad A. Hamilton, George L. Maxwel, George E. Peoples

**Affiliations:** ^1^ Department of Surgery San Antonio Military Medical Center San Antonio Texas; ^2^ National Capital Consortium Fellowship in Gynecologic Oncology Walter Reed National Military Medical Center Bethesda Bethesda Maryland; ^3^ Department of Surgical Oncology The University of Texas MD Anderson Cancer Center Houston Texas; ^4^ Department of Surgery Womack Army Medical Center Fayetteville North Carolina; ^5^ Cancer Vaccine Development Program San Antonio Texas; ^6^ Department of Obstetrics and Gynecology Inova Fairfax Hospital Annandale Virginia; ^7^ Mid‐Atlantic Gynecologic Oncology and Pelvic Surgical Associates Annandale Virginia; ^8^ Inova Health System Inova Schar Cancer Institute Annandale Virginia

**Keywords:** E39, endometrial cancer, FBP, immunotherapy, ovarian cancer, vaccine

## Abstract

**Background:**

E39, an HLA‐A2‐restricted, immunogenic peptide derived from the folate‐binding protein (FBP), is overexpressed in multiple malignancies. We conducted a phase I/IIa trial of the E39 + GM‐CSF vaccine with booster inoculations of either E39 or E39′ (an attenuated version of E39) to prevent recurrences in disease‐free endometrial and ovarian cancer patients(pts). Here, we present the final 24‐month landmark analysis.

**Patients and methods:**

HLA‐A2 + patients receiving E39 + GM‐CSF were included in the vaccine group (VG), and HLA‐A2‐ pts (or HLA‐A2 + patients refusing vaccine) were followed as the control group (CG). VG group received 6 monthly inoculations as the primary vaccine series (PVS) and were randomized to receive either E39 or E39′ booster inoculations. Demographic, safety, immunologic, and disease‐free survival (DFS) data were collected and evaluated.

**Results:**

Fifty‐one patients were enrolled; 29 in the VG and 22 in the CG. Fourteen patients received <1000 μg and 15 received 1000 μg of E39. There were no clinicopathologic differences between VG and CG or between dose groups. E39 was well tolerated. At the 24 months landmark, DFS was 55.5% (VG) vs 40.0% (CG), *P *= 0.339. Patients receiving 1000 μg and boosted patients also showed improved DFS (*P* < 0.03). DFS was improved in the 1000 μg group after treatment of primary disease (90.0% vs CG:42.9%, *P* = 0.007), but not in recurrent patients. In low‐FBP expressing patients, DFS was 100.0% (1000 μg), 50.0% (<1000 μg), and 25.0% (CG), *P* = 0.029.

**Conclusions:**

This phase I/IIa trial reveals that E39 + GM‐CSF is safe and may be effective in preventing recurrence in high‐risk ovarian and endometrial cancer when optimally dosed (1000 μg) to FBP low patients being treated for primary disease.

## 1 INTRODUCTION

Despite some advances in recent years, the 5‐year progression‐free survival for advanced ovarian cancer remains under 20%, highlighting the need for novel therapies.^1^ Immunotherapy is perhaps the most promising of such novel therapies and has garnered a great deal of attention with the recent success of checkpoint inhibitors. Investigators are now seeking other applications of immunotherapy with less toxicity and broader applicability. One such application involves antitumor peptide vaccines derived from tumor‐associated antigens (TAAs), which are capable of inducing tumor‐specific cytotoxic T‐cell (CTL)‐mediated cytolysis with a very low toxicity profile.^2^, ^3^ Additionally, response to these peptide vaccines can stimulate production of memory T cells, offering the promise of long lasting immune surveillance, which should translate into improved disease‐free survival (DFS).^4^ The low toxicity of vaccine therapy coupled with the minimal disease burden make vaccines ideal for the adjuvant setting, where the focus is on recurrence prevention.^5^

Our group has had success with HER2‐directed peptide vaccines^6^ and we are now testing a similar vaccine targeting folate‐binding protein (FBP), which is, in many ways, an ideal target for such a vaccine. FBP is commonly expressed on malignant cells, but rarely in normal tissues,^7^ and tumor cells that do express FBP do so at very high levels, up to 20× higher than normal cells.^3^ Increased levels of FBP expression are also directly associated with a more aggressive disease and, given FBP's role in oncogenesis, tumors cannot readily downregulate its expression.^3^, ^8^, ^9^ Finally, FBP is readily recognized by the immune system, and FBP presentation by dendritic cells leads to tumor‐specific cytokine release and cytolysis.^2^, ^10^ In fact, tumor‐associated lymphocytes (TALs) from HLA‐A2 + ovarian and breast cancer patients recognize FBP peptides without prior in vitro stimulation. Within FBP, the most consistent epitope recognized by TALs is E39 (FBP 191‐199, EIWTHSYKV), an HLA‐A2‐restricted epitope. TALs from E39‐stimulated, HLA‐A2 + patients with FBP‐expressing ovarian cancers not only induced cytolysis in those tumors but also induced cytolysis in other FBP‐expressing epithelial cell‐derived malignancies such as pancreas and colon cancer. Thus, we have created, and are testing the E39 vaccine by administering this peptide with granulocyte macrophage‐colony stimulating factor (GM‐CSF).

Our group has recognized that vaccine‐induced immunity wanes with time, but this can be improved with the use of booster inoculations.^11^ In contrast with this finding, however, there are concerns that repeated stimulation by a single immunogenic TAA may lead to increased tolerance and decreased immunologic memory over time.^12^ For this reason, we have developed an attenuated version of the E39 vaccine, E39′ (EIWTFSTKV, also known as J65), which has been shown in preclinical testing to induce effective FBP‐specific cytotoxicity and has shown efficacy when combined with E39 in an early clinical trial.^13^ Given concerns about repeated stimulation with the same peptide, and the promising preclinical work with E39′, we randomized patients in this trial to boosting with either E39 or E39′ to examine the long‐term immunologic effects.^14^

We have completed a prospective phase I/IIa trial of E39 + granulocyte macrophage‐colony stimulating factor (GM‐CSF) to prevent recurrence in endometrial and ovarian cancer patients at high risk for recurrence after being rendered disease‐free by standard of care therapy. Our previously published interim analysis showed that inoculation with E39 produced a strong, dose‐dependent, in vivo immune response, was well tolerated, and demonstrated some preliminary clinical benefit.^15^ Here, we present the overall 24‐month landmark clinical results based on dose, boosters, and subgroup analyses.

## 2 PATIENTS AND METHODS

### 2.1 Study design

This was a prospective, phase I/IIa trial. Patients were identified who met the following criteria: (a) a diagnosis of ovarian, endometrial, fallopian, or peritoneal cancer; (b) postmenopausal or rendered surgically infertile; (c) completed standard therapies; and (d) no evidence of disease at the time of enrollment. Exclusion criteria included: (a) patients currently receiving immunosuppressive therapy (to include chemotherapy); (b) ECOG > 2; (c) evidence of end‐organ dysfunction; (d) pregnancy; (e) breast feeding; (f) history of autoimmune disease; and (g) involvement in other experimental protocols, except with permission of the principal investigator of the other study.

Patients were enrolled after counseling and consent. Once enrolled, patients were tested for HLA‐A2 status. HLA‐A2 positive patients were offered inclusion in the vaccine group (VG), which was given E39 + GM‐CSF. HLA‐A2 negative patients, as well as HLA‐A2 positive patients who declined vaccination, were followed for disease recurrence as the control group (CG). Vaccinated patients who remained disease‐free 6 months after the primary vaccine series (PVS) were re‐counseled, consented, and randomized to receive booster inoculations of either E39 or E39′.

### 2.2 Folate‐binding protein assessment

To establish FBP status, five de‐identified, paraffin‐embedded slides per histologic type for each enrolled patient were sent to an independent lab for immunohistochemical (IHC) staining with a monoclonal antibody to FBP (FRα‐P). Slides were then scored for FBP (FOLR1) expression levels in large batch by a single pathologist in a blinded manner. Each patient was given an overall score from 0 to 4 based on percentage of sample staining positive. Patients with scores of 0‐1 were characterized as FBP low, while patients with scores of 2‐4 were characterized as FBP high.

### 2.3 Dosing

Patients were enrolled consecutively in a 3 + 3 dose escalation scheme to determine a safe and effective dose, as previously described. 15Briefly, each group received either 100, 500, or 1000 μg of E39. After the third patient in a dose group completed the third inoculation with stable organ function, the next dose group was initiated. The 500 and 1000 μg dose groups were both initially expanded, then the 1000 μg dose group was further expanded based on preliminary findings indicating that this dose may be the most efficacious.^15^ In this final analysis, patients receiving less than 1000 μg (<1000 μg) are compared to those receiving 1000 μg for efficacy analysis.

### 2.4 Vaccine and vaccination series

Peptides were produced commercially by an FDA‐compliant production facility for patient use under IND #12391 for E39 and IND #15305 for E39′. The peptide was purified to >95% before use. Sterility, endotoxin (limulus amebocyte lysate test), and general safety testing was performed. In addition, the manufacturer performed purity/stability testing periodically. Single dose vials were tested for bacterial and fungal contaminants prior to use. The single dose vials were stored in the pharmacy at each institution. The bulk peptide was reconstituted with sterile saline to the following preparations: 100 μg/0.5 mL, 500 μg/0.5 mL, and 1000 μg/0.5 mL. Each of these was mixed with 250 μg/1.0 mL GM‐CSF (Leukine®, Genzyme). This dose of GM‐CSF has been previously determined to be a safe and effective dose, based on our prior work with HER2‐derived peptides.^6^ The combination of peptide and immunoadjuvant had a volume of 1.5 mL, which was administered intradermally at two different sites within 5 cm of each other that drained to the same nodal basin. The primary vaccination series (PVS) consisted of six vaccinations, one given every 21‐28 days and administered in the same lymph node draining area. Treatment began within 28 days of the subject's enrollment in the study.

### 2.5 Boosting

All patients who remained disease free after the PVS were offered booster inoculations. In general, the boosters were given at 6 and 12 months ± 2 weeks after completing the PVS. Patients enrolled prior to the addition of boosters to the protocol, who were more than 6 months out from their PVS, had an individualized booster schedule as determined by the principal investigator. Patients were randomized by a computer‐generated randomization table in a 1:1 allocation ratio to receive either E39 or E39′. Both peptides were dosed at 500 μg and mixed with 250 μg of GM‐CSF. The boosters were given in the same fashion as described above and administered in the same extremity as the PVS.

### 2.6 Toxicity

Patients were monitored closely for 1 hour after all inoculations. Additionally, patients were asked to return to their study site 48‐72 hours after each inoculation for questioning regarding toxicity and to examine and measure local vaccination site reactions. NCI Common Terminology Criteria for Adverse Events, v4.03 was utilized to assess local and systemic toxicity.

### 2.7 Recurrence of disease

Vaccinated and control patients had scheduled follow‐up with their primary oncologists per standard of care. Patients' clinical records were assessed for evidence of recurrence. Disease‐free survival was measured from the date of enrollment. All patients were followed for 2 years.

### 2.8 Statistical analysis

Quantitative data were analyzed by student's *t* test, and categorical data by the chi‐squared test, as appropriate. DFS analysis was done by the Kaplan‐Meier method, and the proportion of subjects who recurred was compared using log‐ranked analysis with significance determined by Mantel‐Cox log rank. Predetermined subset analyses were performed by dose level, pathology, and history of prior recurrence, and level of FBP expression. All statistics were calculated in SPSS (version 22, IBM Corp.). A *P*‐value of <0.05 was considered significant. The data that support the findings of this study are available on request from the corresponding author. The data are not publicly available due to privacy or ethical restrictions.

## 3 RESULTS

### 3.1 Patients

**Figure 1:**
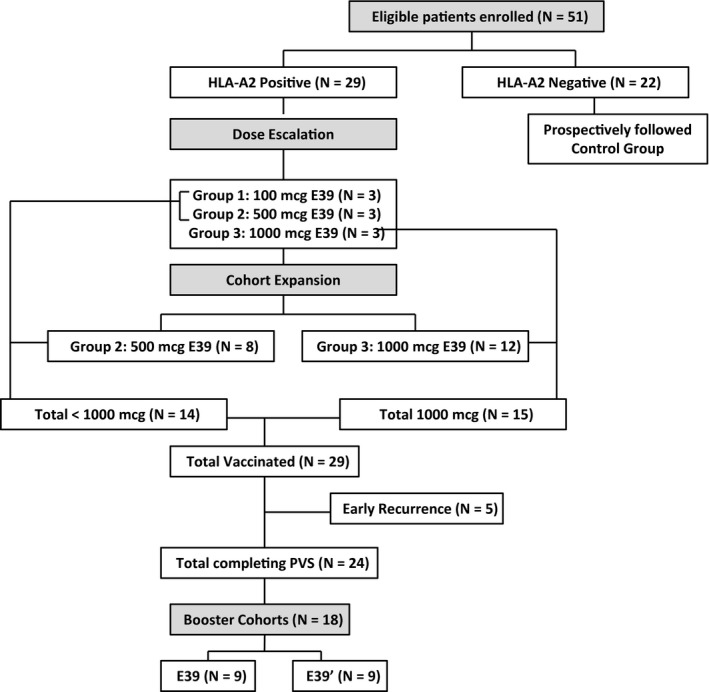
Trial Profile. The final analysis was done on the population as depicted in the consort diagram. HLA‐A2 positive patients were vaccinated while HLA‐A2 negative patients were followed prospectively as controls. The trial initially began as a dose escalation trial with three dosing cohorts. Dosing groups 2 and 3 were expanded. Patients who received <1000 μg per dose (all patients in dosing groups 1 and 2) were compared to those who received 1000 μg per dose (all patients in dosing group 3). All early recurrences occurred in dosing group 2

Fifty‐one patients were enrolled, with 29 in the VG and 22 in the CG (Figure 1). In the VG, 24 patients were enrolled after treatment of primary disease, and 5 patients after treatment of recurrent disease. In the CG, 16 patients were treated for primary, and 6 for recurrent disease. In the VG, five patients recurred before completion of the PVS. Of the remaining 24 patients, 18 continued on to receive at least one booster inoculation (9 patients receiving each of the possible booster peptides, E39 or E39′), and 14 receiving a second booster (7 of each respective peptide). The six patients who did not receive boosters did so because of recurrence or because they declined booster. There were no differences in basic demographic data between dosing groups (Table 1), but, as expected, FBP high patients were more likely to have advanced disease (stage III/IV) than FBP low patients, 84.2% vs 61.1% (*P* = 0.041; Table 2).

**Table 1:** Demographics by dosing

Characteristic	Control	<1000 μg	1000 μg	*P*‐value
Median Age (y)	61	61	57	0.723
(IQR 1‐3)	(53‐64)	(56‐68)	(48‐67)	
Race (%)				0.605
Asian	1 (4.5)	0	1 (6.7)	
Black	0	1 (7.1)	0	
Caucasian	20 (90.9)	12 (85.7)	14 (93.3)	
Hispanic	1 (4.5)	1 (7.1)	0	
Histology (%)				0.531
Ovarian	19 (86.4)	9 (64.3)	11 (73.3)	
Endometrial	3 (13.6)	3 (21.4)	3 (20.0)	
Fallopian	0	1 (7.1)	0	
Peritoneal	0	1 (7.1)	1 (6.7)	
Grade (%)				0.517
1	1 (4.5)	0	2 (13.3)	
2	2 (9.1)	3 (21.4)	1 (6.7)	
3	18 (81.8)	11 (78.6)	12 (80.0)	
FIGO stage (%)				0.435
I	3 (13.6)	1 (7.1)	3 (20.0)	
II	3 (13.6)	0	3 (20.0)	
III	11 (50.0)	11 (78.6)	6 (40.0)	
IV	5 (22.7)	2 (14.3)	3 (20.0)	
Nodal status (%)				0.085
Negative	18 (81.8)	7 (50)	12 (80.0)	
Positive	4 (18.2)	7 (50)	3 (20.0)	
Disease status (%)				0.305
Primary	16 (72.7)	13 (92.9)	11 (73.3)	
Prior recurrence	6 (27.3)	1 (7.1)	4 (26.7)	

**Table 2:** Demographics by FBP expression

Characteristic	FBP hi	FBP lo	*P*‐value
Median Age (y)	58	61	0.753
(IQR 1‐3)	(53‐63)	(54‐67)	
Race (%)			0.367
Asian	1 (5.3)	0 (0)	
Black	0 (0)	0 (0)	
Caucasian	17 (89.5)	18 (100)	
Hispanic	1 (5.3)	0 (0)	
Histology (%)			0.216
Ovarian	17 (89.5)	14 (77.8)	
Endometrial	1 (5.3)	4 (22.2)	
Fallopian	0 (0)	0 (0)	
Peritoneal	1 (5.3)	0 (0)	
Grade (%)			0.366
1	1 (5.3)	2 (11.1)	
2	1 (5.3)	3 (18.7)	
3	17 (89.5)	12 (66.7)	
FIGO stage (%)			0.041
I	1 (5.3)	4 (22.2)	
II	2 (10.5)	3 (16.7)	
III	14 (73.7)	5 (27.8)	
IV	2 (10.5)	6 (33.3)	
Nodal status (%)			0.476
Negative	14 (73.7)	15 (83.3)	
Positive	5 (26.3)	3 (16.7)	
Disease status (%)			0.068
Primary	12 (63.2)	16 (88.9)	
Prior recurrence	7 (36.8)	2 (11.1)	

### 3.2 Toxicity

**Figure 2:**
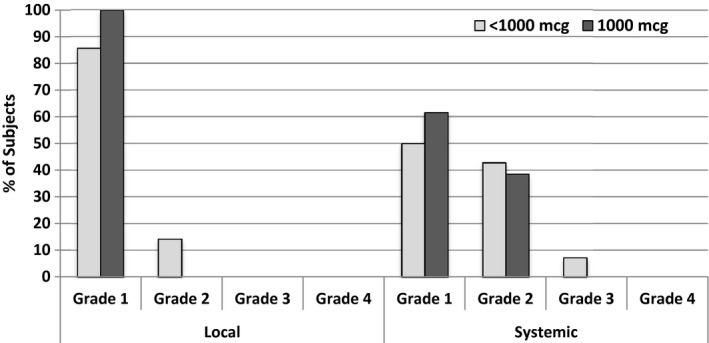
Maximum local and systemic toxicity experienced during the trial per patient divided by dose. The vaccine well tolerated with a single grade 3 systemic toxicity event

Overall, there were no local toxicities greater than grade 2 and no systemic toxicities greater than grade 3 (Figure 2). The toxicity profile was similar between the <1000 and 1000 μg groups. The only grade 3 systemic toxicity occurred in the <1000 μg group (one patient with chest pain/dyspnea that was “possibly related” to study drug). There have been reports of FBP expression in lung tissue in quantitative transcriptomic analysis of human tissue, so it is plausible that this self‐limited toxicity was due to direct immunologic effect of the vaccine.^16^ There were no local or systemic toxicities greater than grade 2 in either booster group.

### 3.3 Disease free survival

#### 3.3.1 Overall

**Figure 3:**
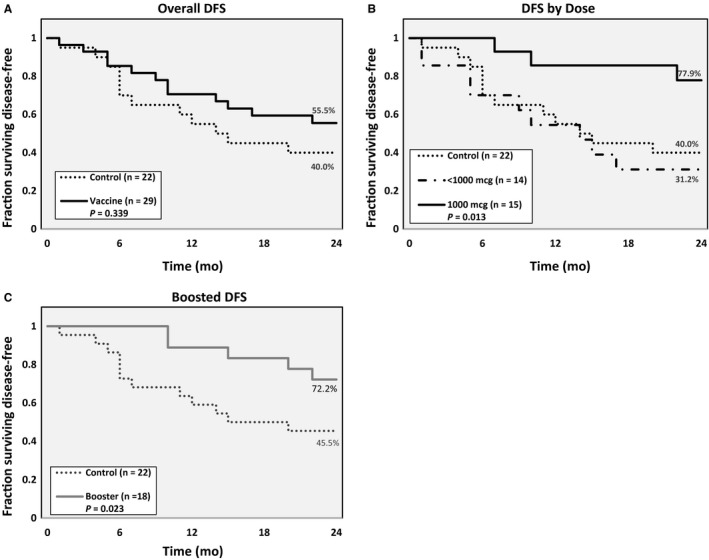
Disease‐fee survival in the (A) overall population, (B) by E39 dosing groups, and by (C) patients who received booster inoculations. Patients had to be disease‐free for 6 months after completion of the primary vaccine series to receive booster inoculations

Our final landmark analysis was performed after the last VG patient reached 24 months DFS, the primary endpoint of the trial. In this final analysis, the DFS was 55.5% in the VG (all doses) vs 40.0% in the CG (*P* = 0.339; Figure 3A). Comparing patients by dose, the DFS was significantly increased in the 1000 μg group (77.9%) over both the <1000 μg group (31.2%) and the control group (40.0%, *P* = 0.013; Figure 3B). Thus, 1000 μg was confirmed to be the optimal dose.

#### 3.3.2 Boosting

DFS was significantly higher in the boosted group than the CG (72.2% vs 45.5%, *P* = 0.023; Figure 3C). Comparing boosted patients to the CG is somewhat biased given that only patients remaining disease‐free 6 months after the PVS were eligible for booster inoculations, but the true intent of boosting was to determine the optimal boosting peptide, E39 vs E39′. There was, however, no significant difference in 24‐month DFS between those boosted with E39 vs E39′ (62.5% vs 77.8%, *P* = 0.068).

#### 3.3.3 Ovarian vs endometrial

Analysis of the VG based on ovarian vs endometrial cancer, 24‐month DFS was not significantly different between the groups (62.5% endometrial vs 47.2% ovarian, *P* = 0.353). However, this trial was not powered for a true comparison of outcomes based on histology, so a difference cannot be realistically excluded.

#### 3.3.4 Primary vs recurrent disease

**Figure 4:**
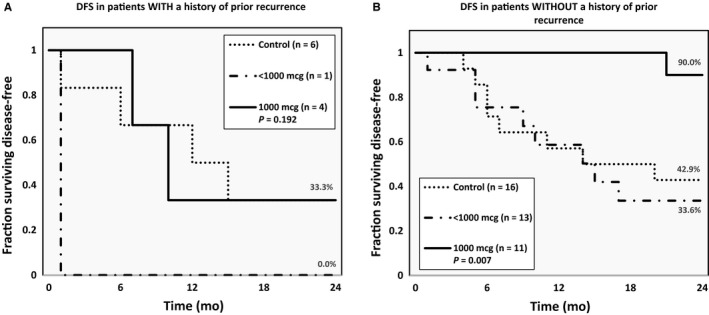
Disease‐free survival in patients rendered disease‐free from (A) recurrent and (B) primary endometrial or ovarian cancer divided by dosing groups

Patients enrolled after being rendered disease‐free (a second time) from a recurrence did not show a significant benefit from vaccination at any dose (*P* = 0.192; Figure 4A). Patients treated for primary disease, however, demonstrated a statistically significant benefit from the 1000 μg dose (90.0%) over both <1000 μg (33.6%) or no vaccination (42.9%) (*P* = 0.007; Figure 4B).

#### 3.3.5 FBP expression

**Figure 5:**
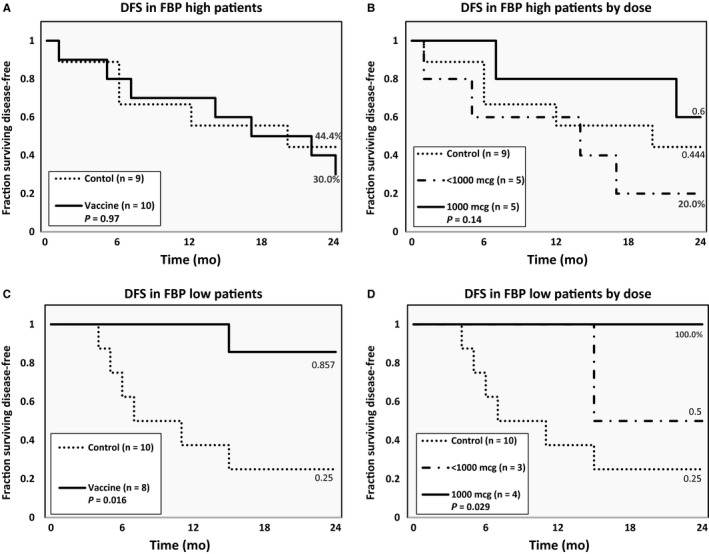
Disease‐free survival in patients by FBP expression levels. Patients with FBP high cancer did not benefit from vaccination in the (A) overall group or by (B) dosing groups. Vaccinated patients with FBP low cancer demonstrated a significant improvement in 24‐month disease‐free survival in the (C) overall population and by (D) the two dosing groups

In FBP high patients, we found no difference in DFS between the VG (30.0%) and the CG (44.4%) (*P* = 0.97; Figure 5A); this lack of benefit persisted when dividing the VG into dosing cohorts (*P* = 0.14, Figure 5B). In the FBP low patients, however, the DFS at 24 months was significantly higher in the overall VG (85.7%) vs the CG (25.0%) (*P* = 0.016; Figure 5C). Moreover, FBP low patients receiving 1000 μg had no recurrences (DFS 100%), which was a significant improvement over patients receiving the <1000 μg (50.0%) and the CG (25%, *P* = 0.029, Figure 5D).

## 4 DISCUSSION

Here, we report the final analysis of the phase I/IIa trial of the E39 peptide vaccine to prevent recurrence in ovarian and endometrial cancer patients at high risk for recurrence. The E39 and E39′ peptides are safe and well tolerated. While there was no significant DFS benefit in the overall cohort, patients treated with the optimal dose of the vaccine did show a benefit. Patients receiving booster inoculations showed a significant increase in DFS as well, though this analysis must be interpreted with caution. Prespecified subgroup analysis showed vaccination was more effective in patients enrolled after treatment of primary disease and those with low FBP expression.

Our group has had previous success producing effective immune responses against the well‐known TAA, HER2, with HER2‐derived peptide vaccines.^6^, ^17^ Given this success, we have targeted another TAA, FBP, with a conceptually similar peptide vaccine. FBP is an excellent target for immunotherapy given its high immunogenicity, minimal expression in normal tissues, significantly increased expression in ovarian and endometrial malignancies, and the correlation between FBP expression and aggressive disease^3^, ^7^, ^8^ The appeal of this TAA as a target has led to the development of other FBP‐directed therapies similar to HER2‐directed therapies. Farletuzumab (MOrAb‐003), a FBP‐targeted monoclonal antibody, was recently tested in a phase III clinical trial, which revealed no significant difference in terms of progression‐free survival overall, but improved progression‐free and overall survival with farletuzumab therapy in patients with CA‐125 levels less than three times the upper limit of normal.^18^ The authors suggested that excessively high levels of CA‐125 may limit the efficacy of farletuzumab by CA‐125‐mediated suppression of natural‐killer cell function, thus inhibiting ADCC.^18^, ^19^ Preclinical work has demonstrated the synergy between a vaccine and mAb directed at the same antigen, and we are completing trials combining trastuzumab and a HER2‐directed vaccine currently.^20^ This concept, if proven effective against HER2, may be applied to FBP with a combination of farletuzumab and our FBP‐derived vaccines.

Our final efficacy analysis of this trial is in line with the interim analysis, with only a trend toward improved DFS in the overall VG, but a significant improvement in optimally dosed patients. We have recently published a trial in which E39 and E39′ were given to patients with breast cancer. In this trial, we found that a lower dose (500 μg) of peptide produced a more effective immune response than the 1000 μg dose. Theoretically, this difference in dose response based on disease type can be explained by the differences in patients' immunity at the time of enrollment. Patients with gynecologic malignancies, compared to breast cancer patients, can be expected to have more immunosuppression as a result of a more aggressive cancer as well as more aggressive chemotherapy. In addition, these gynecologic cancers are known to have higher FBP expression, which means these patients have more exposure to FBP and are more likely to have developed immune tolerance to its derivative peptides, including E39. Thus, in these patients, a larger vaccine dose may be required to induce a meaningful immune response. This theory will need further testing, but the differential response between these two trials points to differing levels of antigen exposure and immune tolerance among subsets of cancer patients, which may have important implications as the field of cancer immunotherapy continues to grow.

While dosing is clearly important to effective immune response, maintenance of this response is key to long‐term efficacy. After observing a waning immunologic response to peptide vaccines in previous trials, we have incorporated booster inoculations in our trials.^6^, ^11^ There is concern, however, that continued stimulation with such immunogenic peptides will lead to overstimulation and, paradoxically, lead to a less effective T‐cell response. The mechanism by which this impaired immunity occurs has not been fully elucidated, but repetitive antigen exposure may lead to the eventual downregulation of CTL response, specifically selecting against clonal populations of effector and memory T cells that are critically important in long‐term immune memory.^21^, ^22^ In preclinical testing, E39′ led to selection of effective T‐cell clonal populations, specifically with a modest cytokine production as measured by IL‐2 and IFN‐*γ*, and more cytolytic activity via effector T cells.^12^ While required for CTL proliferation, elevated levels of IL‐2 have been associated with regulatory T‐cell production, which may impede immune‐mediated clearance of cancer cells.^12^ Similarly, increased IFN‐*γ* is linked to low affinity of the TCR to the HLA‐peptide complex and a subsequently weaker TCR signal which can lead to a premature contraction of effector T‐cell populations.^15^ Given these promising preclinical results with E39′, it was included in our booster series for the current trial. While we were able to show a statistically significant improvement in DFS in patients receiving any type of booster (either E39 or E39′), there was no difference seen between the two peptides. The benefit of boosting in this trial must be interpreted with caution as only patients who remained disease‐free 12 months after enrollment received boosters, creating some inherent bias in the comparison to the control group. We have, however, shown the efficacy of boosting in previous trials, and will continue to incorporate booster inoculations into future studies.^11^, ^13^ With respect to boosting with full strength or attenuated peptides, the current study serves to show the safety of this approach, but additional trials with higher power are needed to elucidate any differences.

While our overall results show promise for this vaccine, predefined subgroup analyses shed light on which patients may show particular benefit. The vaccine is most effective when given to patients who completed therapy for primary disease, with an impressively high estimated DFS (90%) in those treated with 1000 μg dose. Conversely, patients treated for recurrent disease showed no benefit, even at the higher dose. The failure of a peptide vaccine in patients treated for recurrence, who likely had more aggressive disease biology and more immunosuppressive tumor microenvironment, is very much in line with what we already know about peptide vaccines; namely, they are more effective in patients with less disease burden and less aggressive disease.^5^

In any trial utilizing a peptide derived from a specific TAA, it is important to consider the effects of expression level of that TAA. We have previously shown in trials of HER2‐derived peptide vaccines, that the level of HER2 expression can predict vaccine efficacy, with particularly impressive results in patients with HER2 low‐expressing (LE) tumors (HER2 1 + or 2 + by IHC).^6^ In the current trial, we found a very similar effect as E39 vaccination at any dose was not effective in FBP high patients, but vaccination induced a significant improvement in DFS in FBP low patients, with no recurrences at 24 months in the 1000 μg dosed patients. While the mechanism by which high levels of TAA expression lead to a blunted response to vaccination is not fully understood, it is thought to be at least partially due to the development of immune tolerance through in vivo T‐cell anergy after continual antigen exposure.^22^ Additionally, higher TAA expression, especially when these TAA are drivers of malignancy, correlates with more aggressive disease, which is seen in both HER2‐expressing breast cancer and FBP‐expressing malignancies.^8^, ^9^ Whatever the mechanism, an overarching theme runs throughout our subgroup analyses, showing that patients with lower FBP expression and less aggressive disease benefit more from vaccination, which is in line with our previous findings, and likely has broader implications for peptide vaccines as a whole.

## 5 CONCLUSION

This phase I/IIa trial represents the first trial of a single peptide vaccine directed at FBP in endometrial and ovarian cancer to prevent recurrence in clinically disease‐free patients at high risk of recurrence. We have shown that both E39 and E39′ are safe and well tolerated. The optimal dose of E39 in this population is 1000 μg and led to a statistically significant increase in DFS. Booster vaccination may benefit patients, though the dose and type of vaccination (E39 vs E39′) is not yet clear. Subgroup analysis demonstrated the greatest benefit of vaccination for patients treated for primary disease with FBP low expression. E39 and booster inoculation with E39 vs E39′ warrant further investigation in a prospective randomized phase IIb trial utilizing the optimal dosing/boosting regimen and targeting disease‐free primary ovarian cancer patients with low FBP expression levels.

## CONFLICT OF INTEREST

Dr George Peoples has partial inventor rights for the E39 and E39′ vaccines. The view(s) expressed herein are those of the author(s) and do not reflect the official policy or position of San Antonio Military Medical Center, the US Army Medical Department, US Air Force Medical Department, the Department of the Army, the Department of Air Force, Department of Defense or the US Government.

## Data Availability

The data that support the findings of this study are available on request from the corresponding author. The data are not publicly available due to privacy or ethical restrictions.
